# The quality and reliability of short videos about uremia on Bilibili and TikTok in China: A cross sectional study

**DOI:** 10.1097/MD.0000000000047543

**Published:** 2026-02-06

**Authors:** Ying Ying Hang, Hsi Quan Hsu, Man Lu, Lu Bai, Jun Qian

**Affiliations:** a Department of Oncology, Affiliated Hospital of Nanjing University of Chinese Medicine, Nanjing, China; b The First Clinical Medical College, Nanjing University of Chinese Medicine, Nanjing, China; c Jiangsu Collaborative Innovation Center of TCM Cancer Prevention and Treatment, Nanjing, China.

**Keywords:** health communication, information quality, short-video platforms, social media, uremia

## Abstract

Uremia is the end-stage of chronic kidney disease, characterized by the irreversible loss of kidney function and the systemic accumulation of metabolic waste products, which severely impairs patients’ quality of life. In recent years, TikTok and Bilibili have gradually become important sources of health information for patients. This study aimed to evaluate the quality and reliability of uremia-related short videos on these 2 platforms. The top 150 uremia-related videos from the default rankings of each platform were collected, and their general characteristics and engagement metrics were recorded. The global quality scale (GQS) and the modified DISCERN (mDISCERN) instrument were used for evaluation. The Mann–Whitney *U* test and Kruskal–Wallis *H* test were applied to compare differences across groups, and Spearman correlation analysis was conducted to examine associations between video duration, engagement metrics, and quality parameters. A total of 234 videos were included (TikTok: 124; Bilibili: 110). Most videos primarily focused on treatment (28.57%), while critical information, such as epidemiology (3.57%) and diagnostic criteria (11.02%), was underrepresented. The overall median GQS score was 2.00 (interquartile range: 2.00–3.00), and the median mDISCERN score was 2.00 (interquartile range: 2.00–3.00). TikTok videos demonstrated significantly higher user engagement and higher GQS scores compared to Bilibili (median: 3 vs 2, *P* < .05). Videos uploaded by specialists (board-certified physicians, including nephrologists and other medical specialists) achieved the highest GQS and mDISCERN scores (*P* < .05). No significant correlations were found between user engagement metrics and content quality scores (*P *> .05). This study revealed that uremia-related short videos on TikTok and Bilibili were structurally incomplete, with generally suboptimal quality and reliability, though videos uploaded by specialists demonstrated the highest quality. In the future, platforms should strengthen content supervision, improve the structural integrity of video content, and encourage the active participation of specialists to enhance public access to high-quality medical information.

## 1. Introduction

Uremia, the end-stage clinical syndrome of chronic kidney disease, signifies irreversible renal failure that necessitates life-sustaining renal replacement therapy, such as dialysis or kidney transplantation. Globally, both the prevalence and incidence of uremia continue to rise, imposing substantial economic and societal burdens on healthcare systems and affected families.^[1]^ The condition is associated with severe physiological symptoms, multisystem complications, significantly diminished quality of life, and elevated mortality.^[2,3]^ Enhancing public awareness of early symptoms, risk factors, and evidence-based management is crucial for delaying disease progression and improving patient outcomes.^[4]^

In recent years, short-video platforms such as TikTok and Bilibili have become major channels for public health information dissemination, leveraging extensive user bases, algorithmic recommendations, and compelling visual communication.^[5]^ These platforms translate complex medical knowledge into intuitive and accessible content, facilitating health education beyond temporal and spatial constraints.^[6]^ However, the ubiquity of digital media also raises significant concerns regarding the quality and reliability of disseminated information. Due to low creation barriers and the absence of rigorous peer review, health content on these platforms varies widely in accuracy and completeness.^[7]^ Previous studies on short videos related to conditions such as knee osteoarthritis^[8]^ and bipolar disorder^[9]^ have shown that, despite the high viewership and engagement levels of these videos, their scientific quality and reliability are generally suboptimal. Inaccurate or misleading information may foster public misconceptions and potentially delay appropriate diagnosis and treatment.^[10]^ Given that uremia is not only a common manifestation of end-stage chronic kidney disease but is also associated with high disability and mortality rates, imposing a substantial burden on patients’ quality of life and healthcare systems,^[1]^ it is essential to evaluate the quality and reliability of uremia-related videos on short-video platforms.

This study aims to employ standardized evaluation tools to analyze the quality, reliability, and content coverage of uremia-related videos. By providing scientific evidence and systematic analysis, the study seeks to support the development of platform content governance policies, assist healthcare professionals in producing high-quality, evidence-based content, and help the public identify trustworthy health information, thereby promoting the responsible dissemination of digital health knowledge.

## 2. Methods

### 2.1. Search and data collection

**Figure 1.**
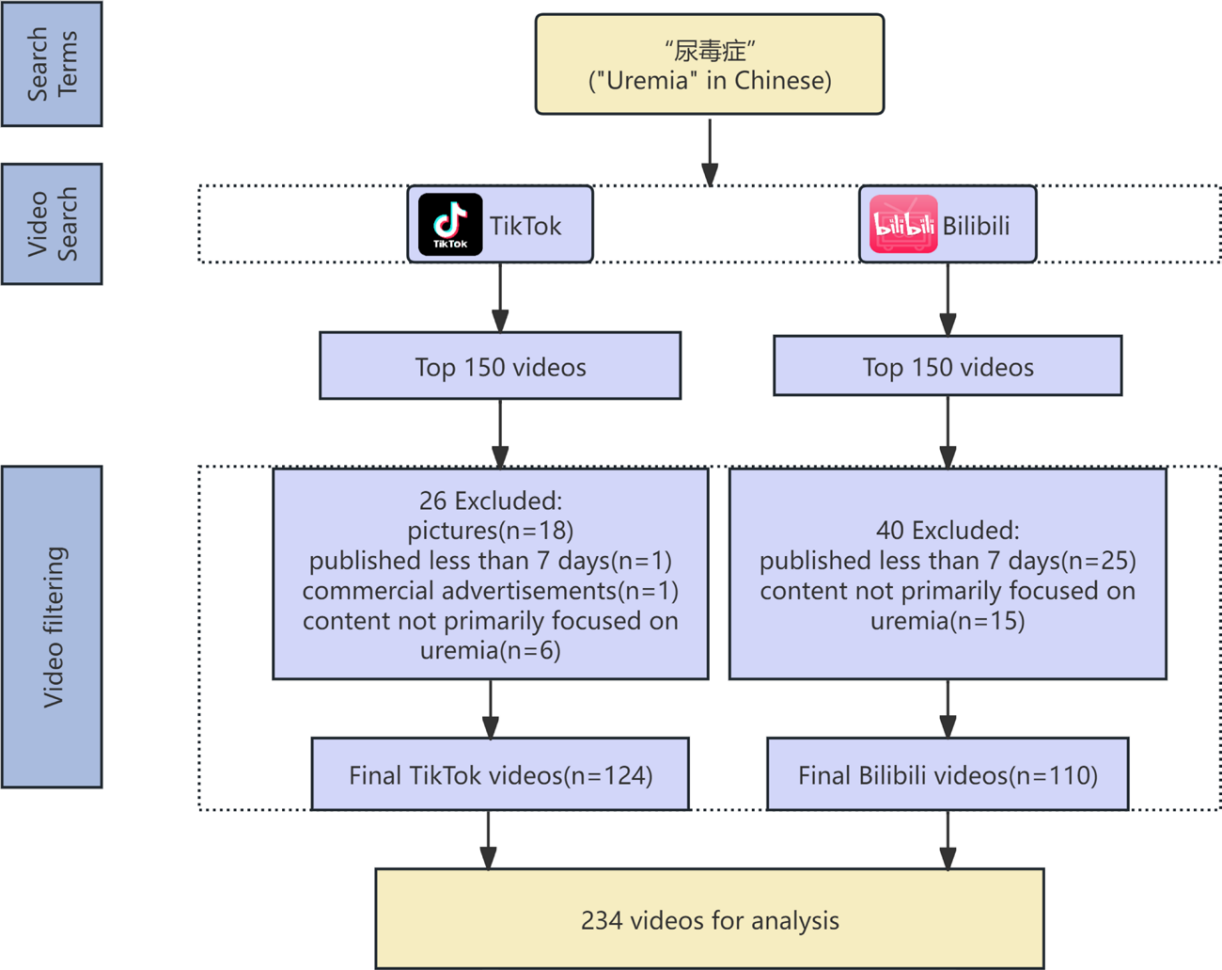
Flowchart of video selection and inclusion process.

This study employed a cross sectional design and was conducted from September 15 to September 18, 2025. We systematically collected short videos related to “uremia” (using the Chinese keyword “尿毒症”) from the TikTok and Bilibili platforms. Only this single keyword was used in the search strategy. To minimize the impact of personalized recommendation algorithms on sample representativeness, all searches were conducted without user login. The top 150 videos from the default ranking on each platform were included for initial screening. The exclusion criteria were as follows: content not primarily focused on uremia (e.g., videos discussing chronic kidney disease without mentioning end-stage manifestations, those focused predominantly on other renal conditions such as nephrotic syndrome or urinary tract infections, or videos where uremia was mentioned only peripherally in discussions about unrelated health topics); videos uploaded <1 week prior; commercial advertisements; duplicate videos; pictures; irrelevant language (e.g., non-Mandarin/English). A detailed collection method is depicted in Figure 1. General information for the included videos (uploaders, likes, collections, shares, comments, and video length) was collected and recorded in Table S1, Supplemental Digital Content, https://links.lww.com/MD/R311.

### 2.2. Uploader characteristics

Based on the identity of the uploaders, they were categorized into 3 groups: specialists: board-certified physicians, including nephrologists and other medical specialists; nonspecialists: uploaders creating health-related content without a medical degree, including health communicators, institutional accounts, and individual users sharing general health information without personal patient experience; patients: individuals explicitly identifying themselves as uremia patients and sharing personal illness experiences or journeys.

### 2.3. Video quality and reliability assessment

Video quality and informational reliability were evaluated using the global quality scale (GQS) and the modified DISCERN (mDISCERN) instrument. The GQS is a widely used tool for assessing the overall quality of health-related videos, scored on a 5-point scale where 1 indicates poor quality and 5 indicates excellent quality.^[11]^ The mDISCERN was used to evaluate the reliability of the health information presented in the videos. It consists of 5 yes/no questions, with each “yes” response scoring 1 point, yielding a total score ranging from 0 to 5.^[12]^ The detailed scoring criteria for the GQS and mDISCERN are presented in Tables 1 and 2, respectively. All videos were independently rated by 2 clinical evaluators (board-certified physicians from the Department of Oncology with research expertise in cancer-related renal injury) who received unified training on the scoring criteria. Any significant discrepancies were resolved by a third senior physician (a senior oncologist with over 10 years of clinical experience and specialized knowledge in renal complications) to ensure specialist input for content-specific disagreements.

**Table 1:** The global quality score (GQS) quality criteria.

Item features	Points
Poor quality; poor flow of the videos; most information missing; not at all useful for patients	1
Generally poor quality; some information listed, but many important topics missing; of very limited use to patients	2
Moderate quality; suboptimal flow; some important adequately discussed, but other information poorly discussed; somewhat useful for patients	3
Good quality and generally good flow; most of the relevant information listed, but some topics not covered; useful for patients	4
Excellent quality and flow; very useful for patients	5

**Table 2:** The modified DISCERN (mDISCERN) quality criteria.

Reliability score	Scores (1 point is given for every yes and 0 points for no)
Is the video clear, concise, and understandable?	0–1
Are valid sources cited?	0–1
Is the content presented balanced and unbiased?	0–1
Are additional sources of content listed for patient reference?	0–1
Are areas of uncertainty mentioned?	0–1

### 2.4. Video content evaluation

Video content was assessed across 6 core dimensions: epidemiological characteristics; disease etiology; clinical symptoms; diagnostic methods; treatment strategies; and preventive measures. Each dimension was recorded and classified according to the completeness and accuracy of the information provided.

### 2.5. Statistical analysis

The distribution of all continuous variables was examined using the Shapiro–Wilk test. All data were non-normally distributed, and continuous variables are presented as median and interquartile range. Group comparisons were performed using the Mann–Whitney *U* test (for 2 groups) or the Kruskal–Wallis *H* test (for multiple groups). The Spearman correlation coefficient (ρ) was used to analyze associations between video metrics (e.g., interaction data, duration) and quality scores. Statistical significance was set at *P* values < .05. All analyses were conducted using R version 4.4.0 (R Foundation for Statistical Computing, Vienna, Austria).

## 3. Result

### 3.1. Video characteristics

A total of 234 short videos pertaining to uremia were included in the analysis from both Bilibili and TikTok platforms. The number of videos from TikTok (n = 124) was slightly higher than that from Bilibili (n = 110). Overall, the quality and reliability of the videos were suboptimal, with a GQS of 2.00 (2.00, 3.00) and an mDISCERN score of 2.00 (2.00, 2.00); the remaining video parameters are presented in Table 3.

**Table 3:** Video characteristics.

Variables	Total (n = 234)
General information	
Video length(s), M (Q1, Q3)	144.00 (65.75, 264.50)
Likes, M (Q1, Q3)	6156.00 (1984.50, 28,656.50)
Collections, M (Q1, Q3)	1236.00 (321.75, 4740.75)
Comments, M (Q1, Q3)	663.00 (192.00, 2733.50)
Shares, M (Q1, Q3)	883.50 (108.00, 13,400.00)
Video quality	
GQS score, M (Q1, Q3)	2.00 (2.00, 3.00)
mDISCERN score, M (Q1, Q3)	2.00 (2.00, 2.00)

### 3.2. Uploader demographics

**Figure 2.**
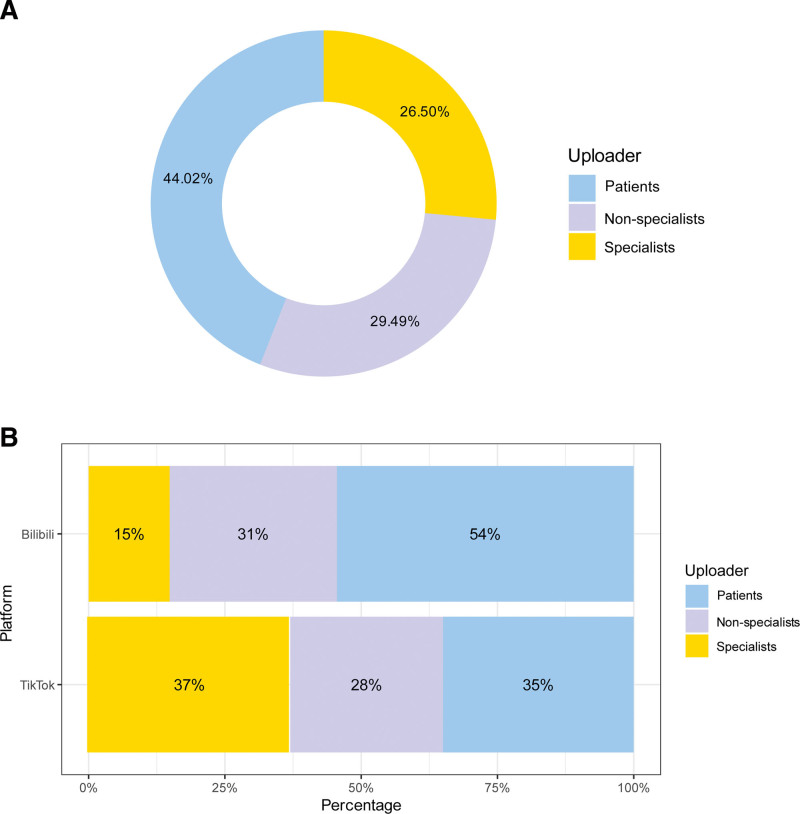
Distribution of video uploaders on Bilibili and TikTok (A) overall distribution of uploader types. (B) Distribution of uploader types on Bilibili and TikTok.

Video uploaders were categorized into patients (44.0%), nonspecialists (29.5%), and specialists (26.5%) (Fig. 2A). The distribution of uploader types differed between platforms (Fig. 2B), with TikTok exhibiting a significantly higher proportion of specialists (37.1%) compared to Bilibili (14.5%), while Bilibili had a markedly greater percentage of patients (34.7%) relative to TikTok (54.5%).

### 3.3. Video content analysis

**Figure 3.**
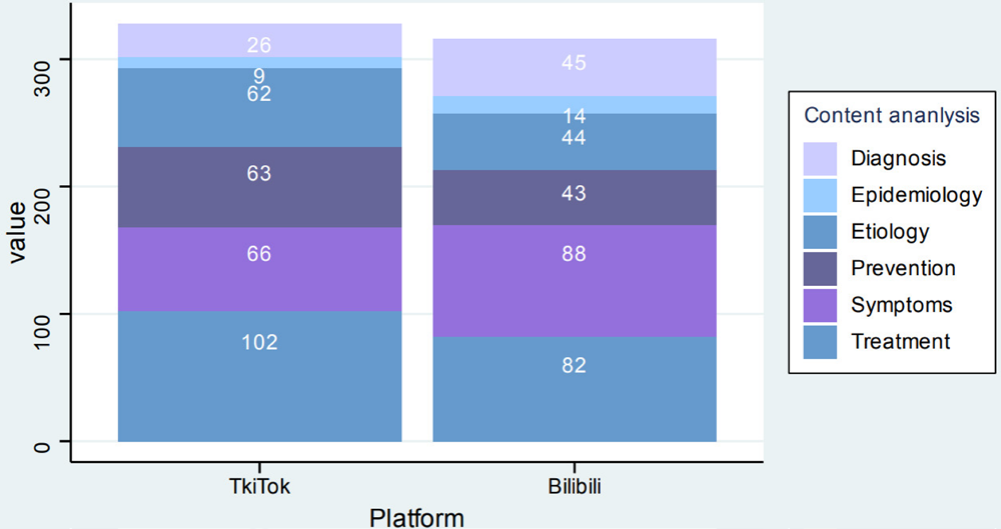
Content coverage of uremia-related topics on TikTok and Bilibili.

Analysis of video content across both platforms revealed that symptoms (23.91%) and treatment (28.57%) were the most extensively covered topics, whereas diagnosis (11.02%) and epidemiology (3.57%) received the least coverage (Fig. 3). Notably, TikTok exhibited a stronger emphasis on “Treatment” content, while Bilibili demonstrated a broader thematic diversity in its coverage.

### 3.4. Cross-platform analysis of video characteristics

**Figure 4.**
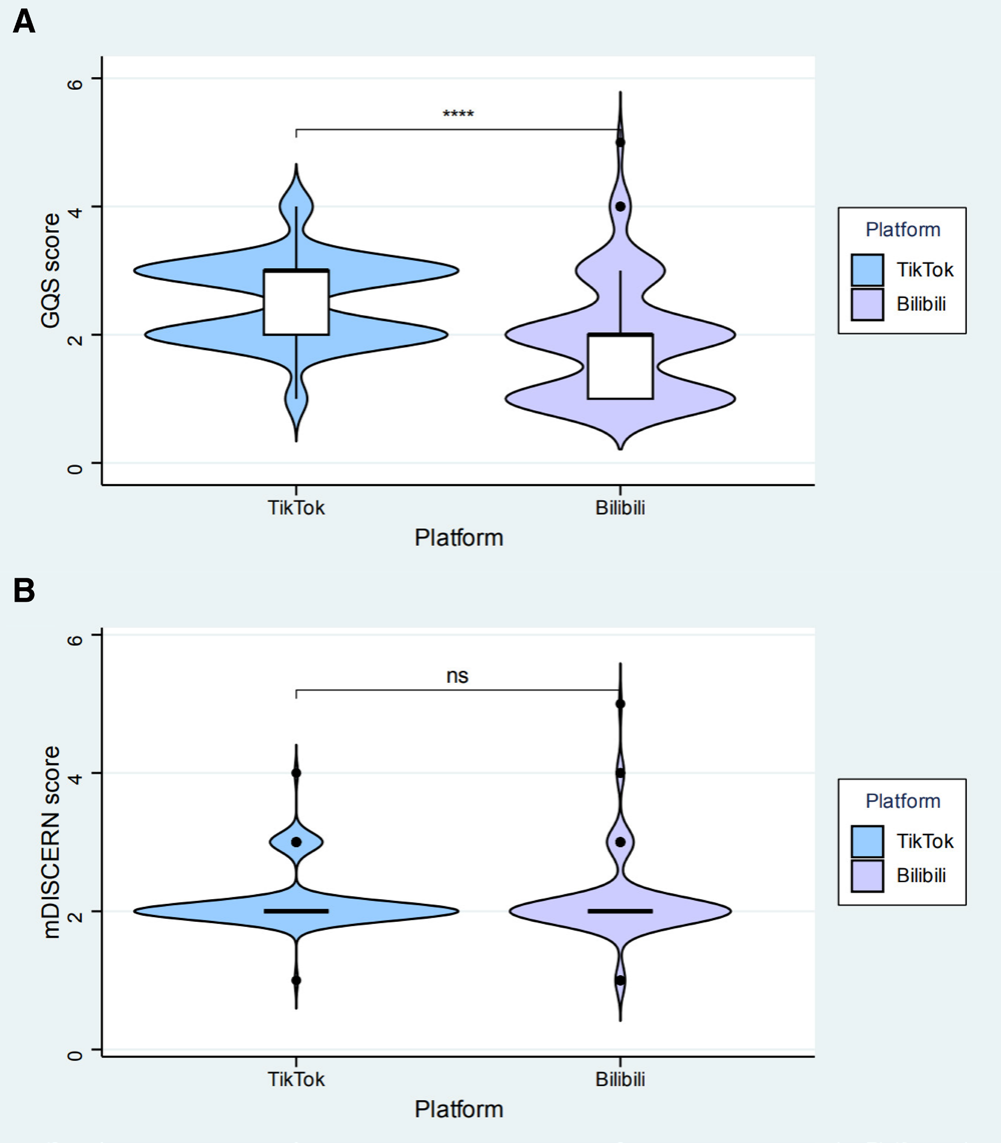
Comparison of quality and reliability scores between TikTok and Bilibili (A) GQS scores. (B) mDISCERN scores.

Significant differences in video characteristics were observed between the 2 platforms (Table 4). TikTok videos demonstrated substantially higher median user engagement metrics compared to Bilibili videos, including likes (15,190 vs 2416), collections (2998.5 vs 407), comments (1802 vs 282.5), and shares (10,448.5 vs 102.5). Conversely, the median video duration was significantly longer on Bilibili than on TikTok (207.5 seconds vs 98.5 seconds). In terms of content quality, the median GQS score was significantly higher on TikTok than on Bilibili (3 vs 2), as shown in Figure 4A. In contrast, no significant difference was observed in the median mDISCERN scores between the 2 platforms (Fig. 4B).

**Table 4:** Comparison of video characteristics between TikTok and Bilibili platforms.

Variables	Bilibili (n = 110)	TikTok (n = 124)	*P*
Video length, M (Q_1_, Q_3_)	207.50 (132.25, 407.00)	98.50 (35.00, 191.00)	<.001
Likes, M (Q_1_, Q_3_)	2416.00 (805.25, 7637.75)	15,190.00 (4624.50, 47,810.50)	<.001
Collections, M (Q_1_, Q_3_)	407.00 (177.00, 1442.75)	2998.50 (808.75, 8398.00)	<.001
Comments, M (Q_1_, Q_3_)	282.50 (98.75, 778.75)	1802.00 (509.25, 7772.50)	<.001
Shares, M (Q_1_, Q_3_)	102.50 (28.00, 448.75)	10,448.50 (1199.75, 69,123.00)	<.001
GQS score, M (Q_1_, Q_3_)	2.00 (1.00, 2.00)	3.00 (2.00, 3.00)	<.001
mDISCERN score, M (Q_1_, Q_3_)	2.00 (2.00, 2.00)	2.00 (2.00, 2.00)	.556

### 3.5. Inter-uploader variability in video features

**Figure 5.**
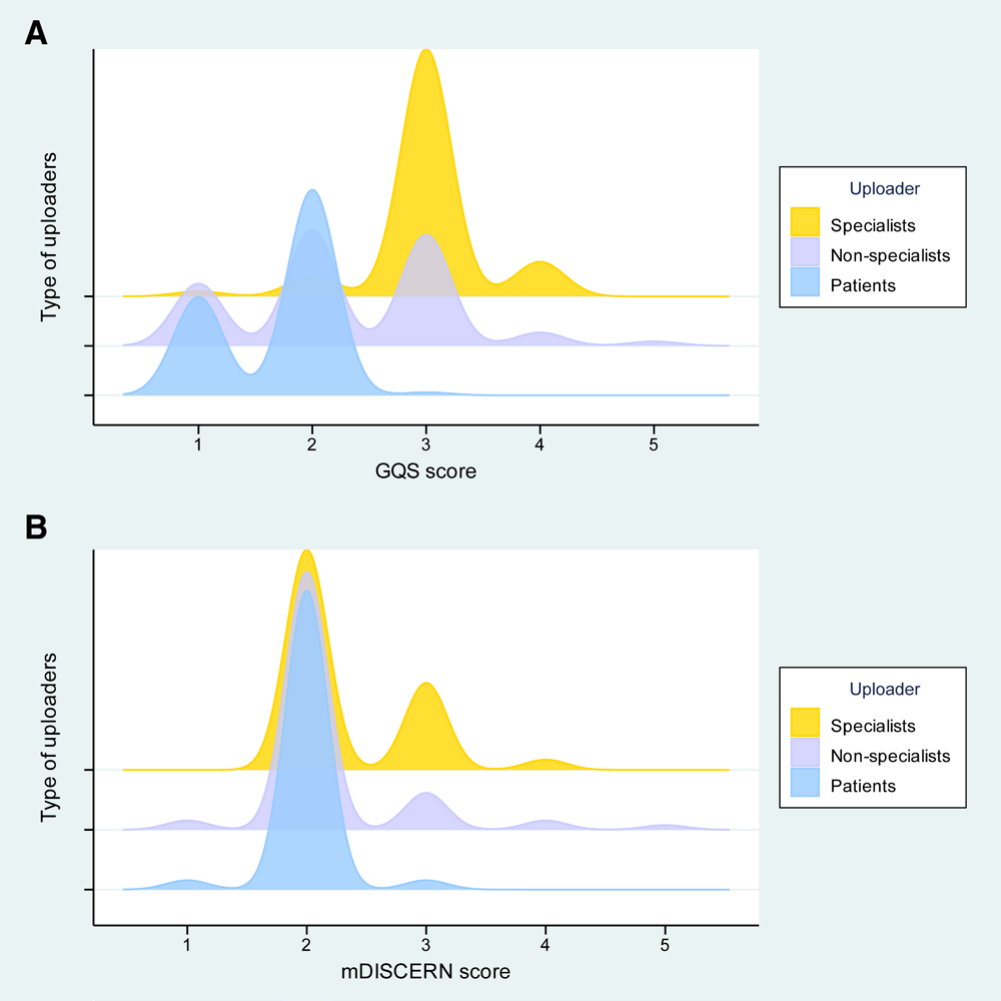
Distribution of quality and reliability scores across different uploader types (A) distribution of GQS scores. (B) Distribution of mDISCERN scores. GOS = global quality scale, mDISCERN = modified DISCERN.

**Figure 6.**
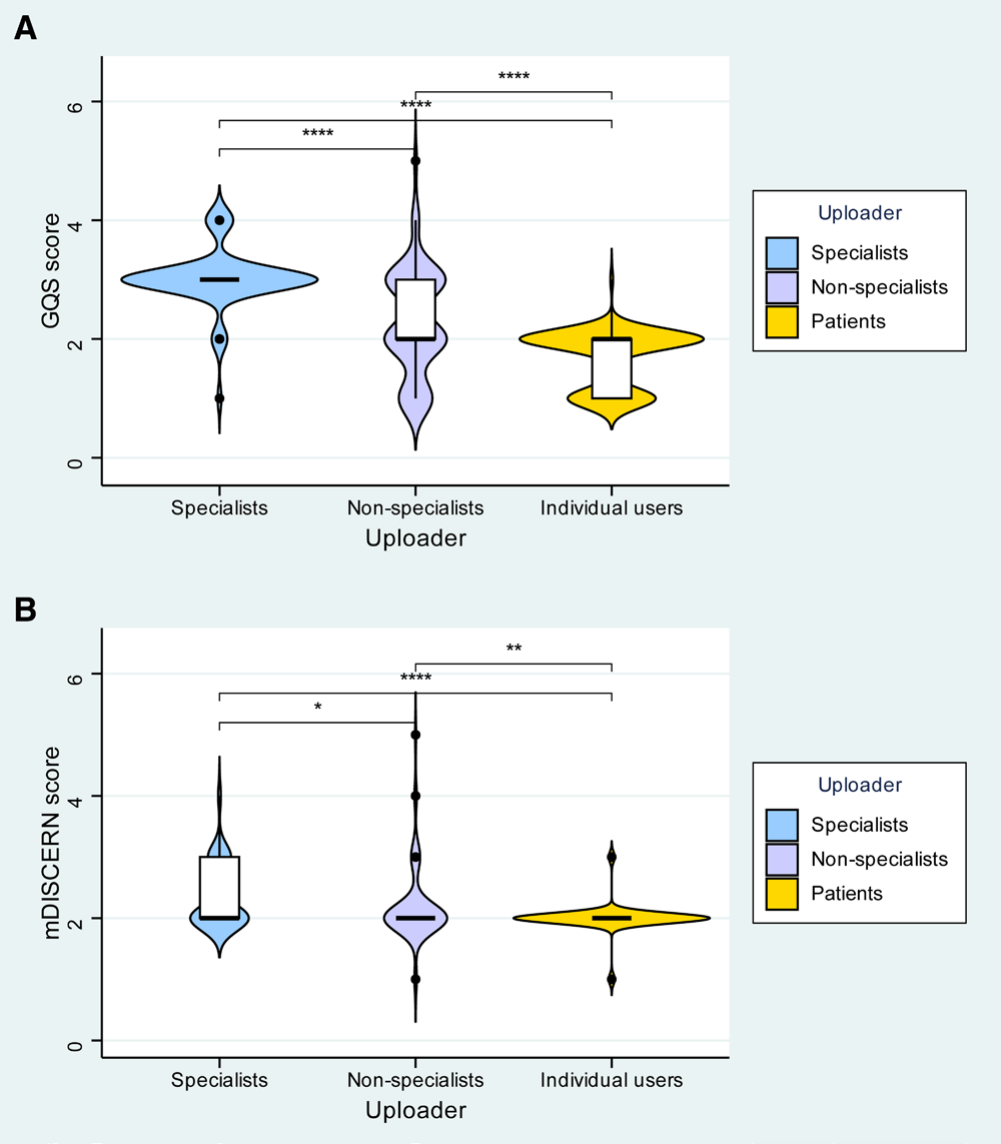
Comparison of quality and reliability scores across different uploader types (A) comparison of GQS scores. (B) comparison of mDISCERN scores. GOS = global quality scale, mDISCERN = modified DISCERN.

Video characteristics varied by uploader type (Table 5). In terms of engagement metrics, videos from specialists received a significantly greater median number of collections and shares. However, no statistically significant differences were observed in median likes or comments across uploader categories. Regarding content quality, videos uploaded by specialists achieved significantly higher median scores in both GQS and mDISCERN compared to those from nonspecialists and patients. Figure 5 illustrates the score distributions within homogeneous uploader groups, with GQS and mDISCERN detailed in panels a and b, respectively; Figure 6A compares the GQS scores across these groups, showing that the specialists achieved the highest scores, followed by nonspecialists, with patients receiving the lowest scores. Figure 6B presents a similar comparison for mDISCERN scores, where specialists again outperformed the other 2 groups.

**Table 5:** Video characteristics stratified by uploader type.

Variables	Total (n = 234)	Patients (n = 103)	Nonspecialists (n = 69)	Specialists (n = 62)	*P*
Video length(s), M (Q_1_, Q_3_)	144.00 (65.75, 264.50)	148.00 (110.00, 239.50)	181.00 (37.00, 509.00)	116.50 (48.00, 215.75)	.189
Likes, M (Q_1_, Q_3_)	6156.00 (1984.50, 28,656.50)	5584.00 (2450.50, 23,568.50)	7058.00 (1277.00, 40,915.00)	7111.00 (2167.75, 23,319.00)	.902
Collections, M (Q_1_, Q_3_)	1236.00 (321.75, 4740.75)	660.00 (212.00, 2044.50)	1937.00 (362.00, 6801.00)	2488.50 (755.75, 6590.00)	<.001
Comments, M (Q_1_, Q_3_)	663.00 (192.00, 2733.50)	649.00 (191.00, 2497.50)	1129.00 (209.00, 5650.00)	576.00 (210.75, 1696.75)	.403
Shares, M (Q_1_, Q_3_)	883.50 (108.00, 13,400.00)	318.00 (40.50, 2832.00)	1165.00 (125.00, 83,821.00)	1596.00 (639.25, 18,461.75)	<.001

### 3.6. Correlation analysis

**Figure 7.**
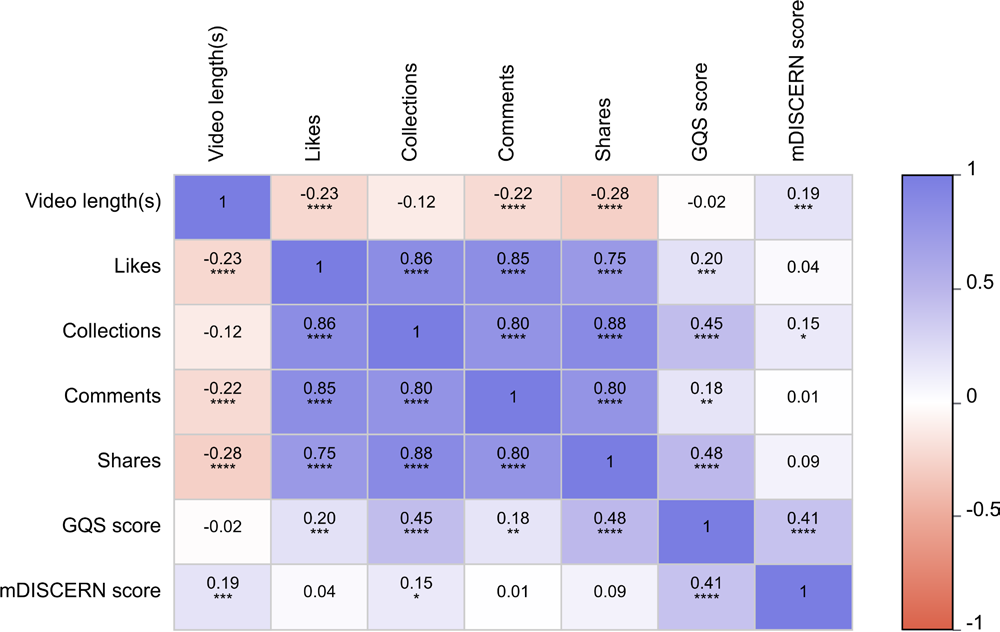
Spearman correlation matrix of video metrics and quality/reliability scores. notation: statistical significance is denoted as follows: *P < .05, **P < .01, ***P < .001. GOS = global quality scale, mDISCERN = modified DISCERN.

Analysis revealed significant negative correlations between video duration and user engagement metrics, including likes (*r* = −0.23, *P* < .001), comments (*r* = −0.22, *P* < .001), and shares (*r* = −0.28, *P* < .001). No significant correlation was observed between video duration and GQS scores (*r* = −0.02, *P* > .05), whereas a weak positive correlation was found with mDISCERN scores (*R* = 0.19, *P* < .001). Furthermore, strong positive correlations were identified among the user engagement metrics: likes showed positive correlations with collections (*R* = 0.86, *P* < .001), comments (*R* = 0.85, *P* < .001), and shares (*R* = 0.75, *P* < .001); collections were positively correlated with comments (*R* = 0.80, *P* < .001) and shares (*R* = 0.88, *P* < .001); comments also demonstrated a significant positive correlation with shares (*R* = 0.80, *P* < .001). Regarding the relationship between engagement metrics and quality scores, collections (*R* = 0.45, *P* < .001) and shares (*R* = 0.48, *P* < .001) showed moderate positive correlations with GQS scores, while likes (*R* = 0.20, *P* < .001) and comments (*R* = 0.18, *P* < .01) exhibited weaker yet statistically significant positive correlations. For mDISCERN scores, only collections demonstrated a weak positive correlation (*R* = 0.15, *P* < .05), while the remaining engagement metrics showed no significant correlations. Finally, a moderate positive correlation was observed between GQS and mDISCERN scores (*R* = 0.41, *P* < .001), as shown in Figure 7.

## 4. Discussion

### 4.1. Principal findings

This study assessed the quality and reliability of uremia-related short videos on TikTok and Bilibili. Significant inter-platform disparities were identified: TikTok videos demonstrated superior user engagement metrics, whereas Bilibili videos were significantly longer. Content uploaded by healthcare professionals scored significantly higher on quality and reliability measures compared to nonprofessional sources, though the latter often garnered higher interaction rates. Critically, video content exhibited substantial gaps in covering essential clinical information, particularly regarding epidemiology and diagnosis. A marked disconnect between the virality and the scientific quality of uremia-related videos was identified. This underscores the urgent need for collaborative efforts among platforms, medical institutions, and the public to enhance the accuracy and accessibility of health information. Future strategies should focus on strengthening cross-platform content moderation, promoting the creation of guideline-based content, and improving public health literacy for discerning high-quality information.

### 4.2. Platform characteristics and dissemination efficiency

Videos on uremia on TikTok exhibited significantly higher levels of engagement, including likes, favorites, comments, and shares, compared to those on Bilibili. This disparity is strongly associated with each platform’s algorithmic design and user behavior patterns.

TikTok promotes brief, high frequency content dissemination. A study on acute pancreatitis video content found that such formats significantly facilitate instant user interaction,^[13]^ a conclusion supported by another study focusing on mammography-related social media engagement.^[14]^ In contrast, although Bilibili accommodates longer video formats, research on stroke-related information dissemination indicates that its content does not necessarily offer greater informational depth, and user engagement remains comparatively conservative.^[15]^ Further analysis of the impact of uploader type revealed that videos created by patients, despite generally lower production quality, received higher levels of engagement. This highlights a distinct “quality-popularity paradox,” consistent with prior findings by Mueller et al.^[16]^ The results suggest that viewers are more attracted to emotionally resonant or narratively compelling content rather than purely scientific accuracy. Therefore, there is an urgent need for platforms to refine their recommendation algorithms to enhance the visibility of scientifically rigorous content and curb the spread of misleading information.

### 4.3. Critical gaps in essential clinical information

Uremia is a severe condition characterized by the complete loss of kidney function. It exhibits high incidence, disability, and mortality rates worldwide.^[2]^ In China, against the backdrop of a rapidly rising prevalence of diabetes and hypertension, the etiology of uremia has shifted toward a predominantly metabolic profile.^[17]^ Early diagnosis and systematic management are essential to delay disease progression and reduce complications such as cardiovascular events.^[2]^ However, this study found that short-video content heavily emphasizes “symptoms” and “treatment,” while providing significantly less coverage of critical topics such as “diagnosis,” “epidemiology,” and “prevention.” This pattern is consistent with previous cross sectional research on the information quality of esophageal cancer-related short videos.^[18]^ Although references to risk factors (e.g., blood pressure and glucose control) are relatively frequent, practical content on staged management and actionable control measures remains insufficient. The lack of diagnostic information is particularly critical and may lead to delays in treatment.^[4,19]^ Such content imbalance, combined with insufficient emphasis on practical risk factor management, may lead patients to overlook early intervention and increase health risks.

### 4.4. Scoring tools reveal platform and uploader health content gaps

The present study revealed that short videos related to uremia on both TikTok and Bilibili exhibited suboptimal overall quality and reliability, a trend consistent with previous studies on cervical cancer-related^[20]^ short videos and diabetes-related^[21]^ content on mainstream platforms. Nevertheless, TikTok videos received significantly higher GQS scores than those on Bilibili, suggesting a moderate advantage in content organization and clarity, which aligns with findings from a cross sectional study on premature ovarian failure-related short videos.^[22]^

Although Bilibili hosts longer video content, greater variability in overall quality was observed. This may be partly attributable to a higher proportion of content generated by nonprofessional creators, which tended to receive lower ratings. The relatively higher GQS scores on TikTok may be associated with its more stringent creator verification processes and enhanced content moderation mechanisms. To improve information credibility and facilitate user discernment, Bilibili could consider implementing stricter authentication procedures for healthcare content creators and introducing a “Verified Health Professional” label to assist users in identifying reliable information more accurately.

Videos uploaded by specialists consistently garnered significantly higher GQS and mDISCERN scores compared to those from nonspecialists and patients, a pattern consistent with previous cross-platform studies on ACL injury prevention content^[23]^ and metabolic disease-related health information.^[22]^ Despite their higher-quality, specialist-generated content did not consistently achieve superior user engagement, indicating a dissemination challenge. The higher proportion of patients on Bilibili raises particular concerns regarding content quality risks, further highlighting the persistent gap between professional and nonprofessional health information online. Platforms should explore strategies like algorithmic boosting or promotional partnerships to enhance the reach of credible content created by HCPs. Concurrently, public health efforts should guide users to prioritize information from certified professional sources to mitigate risks associated with misleading content, a concern similarly observed in studies of sleep-related videos on YouTube^[24]^ and vaccine misinformation online.^[25]^

### 4.5. Quality-popularity paradox in health videos

Spearman correlation analysis revealed strong positive correlations among most user engagement metrics, indicating that high engagement in 1 metric often coincided with high engagement in others, a pattern also observed in studies of bipolar disorder-related content on TikTok.^[9]^ However, contrary to the common assumption, several engagement metrics, particularly collections and shares, showed positive correlations with GQS scores, suggesting that some forms of engagement can co-occur with higher-quality content. Conversely, no such consistent relationship was found with mDISCERN reliability scores, reaffirming the “high interaction ≠ high quality” paradox observed in digital health information, a pattern consistent with findings from research on autism-related videos.^[26]^ Moderate to weak negative correlations were observed between video duration and engagement, suggesting shorter formats may favor dissemination but potentially at the expense of informational depth, a trend aligned with observations from research on Hashimoto’s thyroiditis-related video content.^[27]^

These findings collectively demonstrate that user engagement is a poor proxy for content quality, as metrics such as likes and shares primarily reflect popularity and emotional resonance rather than scientific validity. The dissociation between virality and quality suggests that platform algorithms and user behavior often prioritize entertainment value and algorithmic appeal over educational rigor. Consequently, platforms should develop and integrate evidence-based content evaluation frameworks into their recommendation algorithms to prioritize scientifically accurate information, helping users distinguish between emotionally charged material and reliable health content.

### 4.6. Limitations

This study has several limitations. First, the research focused solely on Chinese platforms, which may limit the external validity of the findings. Second, the cross sectional design captures only a snapshot of content trends, failing to capture the dynamic changes in content over time. Additionally, the small sample size in this study may affect the generalizability and representativeness of the results. Lastly, although validated scales were used, the assessment process inevitably introduced subjectivity.

## 5. Conclusions

This study analyzed the content, quality, and reliability of uremia-related short videos on TikTok and Bilibili. The overall quality and reliability of these videos were suboptimal, with incomplete content coverage, particularly regarding epidemiological features, early screening, and diagnostic criteria. Videos uploaded by specialists achieved the highest GQS and mDISCERN scores. No correlation was found between engagement metrics and video quality or reliability scores. In the future, specialists should be encouraged to participate more actively in uploading educational videos to promote the creation and dissemination of evidence-based, high-quality health content. This study provides practical insights for improving the information quality of digital health platforms and optimizing the dissemination of health information.

## Acknowledgments

The authors express their gratitude to the video uploaders for their contributions to public health.

## Author contributions

**Conceptualization:** Ying Ying Hang.

**Writing – original draft:** Ying Ying Hang.

**Formal analysis:** Hsi Quan Hsu.

**Methodology:** Hsi Quan Hsu.

**Data curation:** Man Lu.

**Investigation:** Man Lu.

**Writing – review & editing:** Lu Bai.

**Project administration:** Jun Qian.

## Supplementary Material


